# Dual RNA-Sequencing of *Eucalyptus nitens* during *Phytophthora cinnamomi* Challenge Reveals Pathogen and Host Factors Influencing Compatibility

**DOI:** 10.3389/fpls.2016.00191

**Published:** 2016-03-02

**Authors:** Febé E. Meyer, Louise S. Shuey, Sitha Naidoo, Thandekile Mamni, Dave K. Berger, Alexander A. Myburg, Noëlani van den Berg, Sanushka Naidoo

**Affiliations:** ^1^Department of Genetics, Forestry and Agricultural Biotechnology Institute, Genomics Research Institute, University of PretoriaPretoria, South Africa; ^2^Department of Plant Science, Forestry and Agricultural Biotechnology Institute, Genomics Research Institute, University of PretoriaPretoria, South Africa

**Keywords:** plant defense, RNA-seq, *Eucalyptus nitens*, pathogenesis related genes, crinkler

## Abstract

Damage caused by *Phytophthora cinnamomi* Rands remains an important concern on forest tree species. The pathogen causes root and collar rot, stem cankers, and dieback of various economically important *Eucalyptus* spp. In South Africa, susceptible cold tolerant *Eucalyptus* plantations have been affected by various *Phytophthora* spp. with *P. cinnamomi* considered one of the most virulent. The molecular basis of this compatible interaction is poorly understood. In this study, susceptible *Eucalyptus nitens* plants were stem inoculated with *P. cinnamomi* and tissue was harvested five days post inoculation. Dual RNA-sequencing, a technique which allows the concurrent detection of both pathogen and host transcripts during infection, was performed. Approximately 1% of the reads mapped to the draft genome of *P. cinnamomi* while 78% of the reads mapped to the *Eucalyptus grandis* genome. The highest expressed *P. cinnamomi* gene *in planta* was a putative crinkler effector (*CRN1*). Phylogenetic analysis indicated the high similarity of this *P. cinnamomi CRN1* to that of *Phytophthora infestans*. Some CRN effectors are known to target host nuclei to suppress defense. In the host, over 1400 genes were significantly differentially expressed in comparison to mock inoculated trees, including suites of pathogenesis related (*PR*) genes. In particular, a *PR-9* peroxidase gene with a high similarity to a *Carica papaya PR-9* ortholog previously shown to be suppressed upon infection by *Phytophthora palmivora* was down-regulated two-fold. This *PR-9* gene may represent a cross-species effector target during *P. cinnamomi* infection. This study identified pathogenicity factors, potential manipulation targets, and attempted host defense mechanisms activated by *E. nitens* that contributed to the susceptible outcome of the interaction.

## Introduction

Species of the Oomycete genus *Phytophthora* are the most economically important pathogens of plants worldwide (Zentmyer, 1983; Erwin and Ribeiro, 1996). Of critical concern to the forestry industry is *Phytophthora cinnamomi* Rands, one of the most pathogenic and damaging species that affects agriculture, forestry, and native forests worldwide (Linde et al., 1999; Brasier, 2008; Hansen, 2008; Oβwald et al., 2014). In *Eucalyptus, P. cinnamomi* has threatened the productivity of plantations (FAO, 2007; Wingfield et al., 2011) and caused extensive damage to native ecosystems including *E. marginata* forests in Western Australia (Podger et al., 1967; Burgess et al., 1999) and native vegetation of the Western Cape of South Africa (Vonbroembsen and Kruger, 1985). Eucalypt plantations contribute significantly to the economy of South Africa (Godsmark, 2009). Susceptible cold-tolerant *Eucalyptus* plantations have been affected by various *Phytophthora* species to such an extent that some valuable species such as *E. fastigata* and *E. fraxinoides* are no longer cultivated (Linde et al., 1994; Wingfield and Kemp, 1994). *Eucalyptus nitens* is considered resistant to *P. cinnamomi* in its native environment but succumbs to the pathogen in plantations (Cahill et al., 2008). *P. cinnamomi*, an introduced stramenopile pathogen (Adl et al., 2005, 2012) severely affected stands of *E. nitens* in South Africa (Maseko, 2010).

Plant-pathogen interactions in forest systems have not been as well studied at a genomic level as it has for herbaceous crops. The recent availability of the genome sequence of *E. grandis* (Myburg et al., 2014) has provided a valuable resource for transcriptomic studies to dissect defense responses in related *Eucalyptus* species. RNA sequencing (RNA-seq) has contributed knowledge towards several host responses during pathogen challenge (Xu et al., 2011, 2012; Dowen et al., 2012; Martinelli et al., 2012; Savory et al., 2012; Tremblay et al., 2012). Unlike previous probe based methods which require separation of host and pathogen cells, RNA-seq has allowed the study of both host and pathogen transcriptomics simultaneously. This technique, known as dual RNA-seq (reviewed in Westermann et al., 2012) allows the detection of minute amounts of pathogen RNA. It does not require predesigned species specific probes and is more sensitive than the previous methods of microarrays and northern blotting (Kunjeti et al., 2012; Tierney et al., 2012; Westermann et al., 2012; Camilios-Neto et al., 2014; Choi et al., 2014; Hayden et al., 2014). Pathogen RNA-seq data can be further mined for clues to pathogenicity (ability to cause disease) and virulence (degree of damage or pathology) factors based on functional genetics studies conducted in various host-pathogen interactions. The plant-host interactions database provides such data for comparative analysis (Winnenburg et al., 2006).

An enhanced understanding of the defense response of plants to *P. cinnamomi* will facilitate the production of resistant plants (Eshraghi et al., 2014a). Several RNA-seq host response studies to *Phytophthora* spp. have been undertaken to date (Kunjeti et al., 2012; Ali et al., 2014; Chen et al., 2014). The host responses of raspberry to *P. rubi*, and those of potato tubers to *P. infestans*, were successfully profiled using RNA-seq (Ward and Weber, 2012; Gao et al., 2013). This technique has also been applied to a native forest system, with the response of the oak *Notholithocarpus densiflorus* to *P. ramorum* elucidated (Kunjeti et al., 2012; Ward and Weber, 2012; Gao et al., 2013; Ali et al., 2014; Chen et al., 2014; Hayden et al., 2014).

The genome sequence of *P. cinnamomi var. cinnamomi* is currently available as a draft assembly of 77.97 Mbp and a sequence read coverage depth of 69.6x (Reeve, 2012, unpublished; JGI Project identity: 1003775). This is an invaluable resource that provides insight into pathogenicity determinants in this species. This has been demonstrated for other *Phytophthora* species sequenced genomes (e.g., *P. infestans, P. sojae, P. ramorum*, reviewed in Jiang and Tyler, 2012).

*Phytophthora* species are able to manipulate their hosts to their own advantage. For example, *P. infestans* manipulates its host to suit its life-style by suppressing the hypersensitive response (HR) in potato during its biotrophic phase, then manipulating the induction of HR during the necrotrophic phase (Bos et al., 2010; Gilroy et al., 2011). This type of manipulation could be a trend in other *Phytophthora* interactions (Belhaj et al., 2009; Porter et al., 2009). Effectors excreted during this interaction are primarily crinklers (CRN), a family of proteins expressed in all plant pathogenic oomycetes, and RxLRs which are confined to only *Phytophthora* species (Stam et al., 2013b; Chen et al., 2014).

Host defense responses are mediated through different mechanisms, and the timing and degree to which these are activated could determine the outcome of the interaction between a plant and pathogen (Tao et al., 2003). A factor driving compatible interactions has been revealed by several studies to involve a mass down-regulation of defense genes (Schlink et al., 2010). Pathways associated with these genes include the defense hormone salicylic acid (SA) which is associated with biotrophic defense responses, jasmonic acid (JA), and ethylene (ET) which are associated with necrotrophic defense responses, and abscisic acid (ABA) which is associated with abiotic stress as well as pathogen defense (Bari and Jones, 2009). The host responses elicited by *P. cinnamomi* have been studied in different woody species at various levels including anatomical, physiological, biochemical, and molecular levels (recently reviewed in Oβwald et al., 2014). Some of the major findings in these studies highlight the importance of correctly regulated HR and reactive oxygen species (ROS) and synthesis of phenylpropanoid pathway-related substances such as flavonoids, gibberellic acid (GA) and lignin. *PR*-*1* and *PR*-*5* feature in several interactions as possible resistance factors.

Multiple pathogenesis related (*PR*) gene classes are differentially regulated against *Phytophthora* and are thought to be important for successful defense (Moy et al., 2004; Schlink, 2009; Attard et al., 2010). The over-expression of specific *PR* genes have conferred tolerance against various *Phytophthora* spp. (Alexander et al., 1993; Fagoaga et al., 2001; Sarowar et al., 2009; Pushin et al., 2010; Acharya et al., 2013; He et al., 2013).

The *E. nitens–P. cinnamomi* interaction provided a novel system to study a compatible host-pathogen interaction using a dual RNA-sequencing approach. The aim of this study was to (i) discover pathogenicity factors produced by *P. cinnamomi* (ii) to determine the *E. nitens* defense response to the pathogen, and (iii) to identify host genes that could potentially be suppressed by the pathogen to promote susceptibility. We observed, among other responses, high expression of a putative *P. cinnamomi* crinkler effector *in planta* and the down-regulation of a *PR*-9 gene, which may represent a common host effector target in *E. nitens*; two factors possibly contributing to the susceptible outcome of the interaction.

## Methods

### Inoculated plant material

*E. nitens* seedlings were obtained from parents that were part of a third generation commercial breeding program (Sappi Forests Research, Shaw Research Centre, KwaZulu-Natal, South Africa) The criterion for selection was based on wood density gain. An MLRelate analysis, using the microsatellite markers developed by Faria et al. (2010), determined that the individuals from the Sappi breeding population showed higher relatedness to each other than those within the natural Australian *E. nitens* population (Melissa Reynolds, Forest Molecular Genetics, University of Pretoria). Previous sampling in *E. nitens* stands in South Africa have shown root rot due to *P. cinnamomi* (Maseko, 2010).

The *E. nitens* seedlings were grown in pine-bark seedling mix until stem thickness was >0.5 cm (~1 year), then stem inoculated with *P. cinnamomi* (CMW26310, Forestry and Agricultural Biotechnology Institute culture collection). A 4 mm cork borer and mycelial plug on cV8 agar [cV8A; modified from Erwin and Ribeiro (1996): 200 ml/L V8 juice (Campbell Soup Company, Camden, New Jersey), 20 g/L CaCO_3_ (Merck), 20 g/L agar)] was used for inoculations. Mock-inoculated plants were treated identically to infected trees, with a sterile cV8 agar plug. Inoculation sites were covered with damp sterile cheesecloth, tinfoil, and Parafilm (Parafilm, Chicago, IL).

Stems were inoculated at two sites, 10 cm apart. In the attempt to represent the typical responses to *P. cinnamomi* in commercially grown *E. nitens*, for both the mock-inoculated control and inoculated trees, stem tissue was harvested from 18 trees (which consisted of three pooled biological replicates of six trees each) at 5 days post inoculation (dpi). Three centimeters of stem tissue was harvested per inoculation site, with 1.5 cm of stem tissue below and above the center of the site. Harvested material was immediately frozen in liquid nitrogen.

Since sampling was destructive, nine extra trees were used to observe symptom development for 6 weeks following inoculation. Beneath-bark lesions were measured in other trials to statistically validate the effectivity of inoculation. For these lesions, a Shapiro-Wilk test was performed in GraphPad Prism 6 (Motulsky, 1999) and the non-parametric Mann-Whitney test was used to assess significance at a 95% confidence level.

### Microscopy

#### Preparation of plant material

Approximately 1.5–2 cm of stem tissue surrounding the inoculation site of *P. cinnamomi* inoculated and mock-inoculated *E. nitens* tissue was harvested in triplicate at 24 h post inoculation (hpi), 48 hpi, 96 hpi, and 1 week post inoculation (wpi). The harvested tissue was fixed in formalin-acetic acid-alcohol [FAA; 100 ml/L formalin, 50 ml/L glacial acetic acid, 500 ml/L 95% ethanol]. For confocal microscopy, thin longitudinal- and cross-sections were made of stem tissue away from the immediate vicinity of the inoculation site. These sections were stained in 0.01% Calcofluor white fluorescent brightener 28 (Sigma-Aldrich®).

#### Visualization

Representative stem tissue samples from each time-point were visualized under a Stemi SV6 stereo microscope (Zeiss, Munchen, Germany). Images were captured using an AxioCamMRc digital camera (Zeiss) and Axiovision 4.7 software (Zeiss). The presence of hyphae inside the sampled tissue at various time-points was verified through use of a confocal laser scanning microscope (CLSM 510 Meta, Zeiss). To view Calcofluor-stained tissue, a wavelength of 405 nm was used, and a 543 nm wavelength was used to visualize autofluorescence. LSM Image Browser v4.2.0.121 (Zeiss) was used to view images generated from the confocal microscopy.

#### RNA extraction and quality analysis

RNA was isolated using a modified cetyl-trimethyl-ammonium-bromide (CTAB) method (Naidoo et al., 2013). On-column DNAse treatment with 10 units DNaseI (Fermentas, Ontario, Canada) and RNA purification was performed with the RNeasy® Mini kit (Qiagen, Valencia, California). Quality analysis was done on a 2100 Bioanalyzer (Agilent, Santa Clara, California).

#### RNA sequencing

Approximately 20 μg total RNA for each sample was submitted for sequencing at Beijing Genomics Institute (BGI, Beijing, China). Sequencing of mRNA was performed with random fragmentation of the mRNA and adapter ligation. Fifty bp paired end reads were obtained using an Illumina HiSeq 2000 (Illumina, San Diego, CA).

### Bioinformatic analysis

#### Quality analysis and filtering

Adaptors, low quality reads, and reads with more than 10% unknown nucleotides were removed from the dataset by BGI. The Galaxy platform (Giardine et al., 2005; Blankenberg et al., 2010b; Goecks et al., 2010) was used to analyze and process the RNA-seq reads. FASTQ Groomer (Blankenberg et al., 2010a) and FASTQC (Babraham Bioinformatics, http://www.bioinformatics.babraham.ac.uk/projects/fastqc/) were used to format reads and to assess read quality.

#### Mapping and transcript expression analysis of the host

Mapping to the *E. grandis* v1.1 genome was done with Bowtie (Langmead et al., 2009) and TopHat v1.3.1 (Trapnell et al., 2009), allowing for 2 bp mismatches per 50 bp read and a maximum intron length of 10,000 bp. Mapping statistics were verified using SAMtools flagstat (Li et al., 2009). Assembly of mapped reads and calculation of expression values of predicted *E. grandis* transcripts as fragments per kilobase of transcript per million fragments (FPKM) was performed by Cufflinks (Trapnell et al., 2010). Significant differential expression analysis was done with Cuffdiff (Trapnell et al., 2010), where parameter settings were changed to allow for a minimum alignment count of 1000, a false discovery rate of 0.05, as well as quartile normalization and bias correction. Additional parameter settings were set to an average fragment length of 49 bp and a standard deviation of 10 bp for the fragment lengths.

#### Mapping and transcript expression analysis of the pathogen

Reads from the three uninoculated samples and the three inoculated samples were mapped to the *P. cinnamomi* var *cinnamomi* draft genome downloaded from the Joint Genome Institute (Reeve, 2012, unpublished; JGI Project identity: 1003775). The genome assembly, based on Illumina, 454 and Sanger sequencing, is 77.97 Mbp and the sequence read coverage depth is 69.6x (Grigoriev et al., 2012). Mapping was performed as described above and assembly of reads and FPKM value were generated using Cufflinks. Any genes or transcripts showing an FPKM value above 100 in the control samples were considered conserved eukaryotic genes and removed from further analysis. Remaining genes or transcripts which showed FPKM values >100 in the three inoculated samples with a coefficient of variation < 0.2 were considered further. Approximately 280 genes satisfied this criteria and were subsequently analyzed using the pathogen host interactions (PHI) database (http://www.phi-base.org/ Winnenburg et al., 2006) to determine pathogenicity or virulence factors expressed *in planta* using a local BLASTP search in CLCBio main workbench (version 6.1; Qiagen). The database was downloaded in July 2014 and hits with the lowest *e*-value were considered. Selected genes were also subjected to Blast2GO® analysis to annotate the gene models.

#### Gene ontology over-representation analysis of host transcripts

Microsoft Excel 2007 (Microsoft, Redmond, WA) was used to match significantly differentially expressed *E. grandis* v1.1 gene and transcript models to TAIR10 and TAIR9 identifiers based on a reciprocal BLAST analysis. The significantly differentially expressed genes (and transcripts for which the representative gene model was not differentially expressed) with TAIR10 putative orthologs were divided into up- and down-regulated datasets, which were analyzed for gene ontology (GO) over-representation in BiNGO v2.44 (Maere et al., 2005) using the Cytoscape v2.8.3a platform (Shannon et al., 2003). Over-representation was evaluated against the *Arabidopsis thaliana* genome in the categories for “biological processes,” “molecular function,” and “cellular component.” A hypergeometric test with a Benjamini & Hochberg FDR correction of 0.05 was used. Understanding of biological pathways was aided by MapMan v3.5.1R2 (Thimm et al., 2004).

#### RT-qPCR

Genomic contamination in total RNA samples was removed by treating extracted total RNA samples with RNase-free DNaseI enzyme (Qiagen Inc., Valencia, CA). Total RNA samples were then purified using the RNeasy® MinElute Kit (Qiagen Inc.) and subsequently analyzed using a Bio-Rad Experion automated electrophoresis system (Bio-Rad Laboratories, Hercules, CA, USA), to determine RNA integrity. The Improm-IITM Reverse Transcription System (Promega, Wisconsin, USA) was used to synthesize first strand cDNA from purified RNA samples. Primers were designed using Primer Designer 4 v4.20 (Sci Ed Central, Cary, North Carolina, USA). Primer pairs are indicated in Table 1.

**Table 1:** **Primer sequences for *Eucalyptus nitens* RT-qPCR target and reference genes**.

Eucalyptus ID^*^	**Gene name**	**Forward primer**	**Reverse primer**
Eucgr.B03520	*EgrWRKY75*	AAGCGCCAGCAGCGGTGGATGAGAA	TGCAGCCGTGGAACGTGTCAACGGTA
Eucgr.F02181	*EgrLRR-RLK7*	TTGGTGAATCTCTGGCGACTTGAGC	GACAGATTGACGAGAGCCTCTGGAACT
Eucgr.H02533	*EgrNRT2.5*	TGTCCGAATGGAGCGACAAGGAGAA	ACACGGTGCACGAGTACATGAACAG
Eucgr.I01495	*EgrPR-3*	GTATTGCTCTCCTAATCC	CATTGCCCGTAGTTATAG
Eucgr.J01100	*EgrMLO*	GTCAAGAGGTCATTAGAAG	TAGAAGCAAGAAGATAACG
Eucgr.I01779	*EgrARF*	TGCGTACCGAGTTGTTGAGG	GTTGCACAGGTGCTCTGGAT
Eucgr.B02864	*EgrFBA*	TGAAGACATGGCAAGGAAGG	GTACCGAAGTTGCTCCGAAT
Eucgr.G01186	*EgrTUB*	TGAGGTCTTCTCGCGCATTG	AGAGATCTGGCGCAGACAC

* *Eucalyptus grandis identities according to www.phytozome.net*.

Real-time quantitative reverse transcriptase PCR (RT-qPCR) was conducted according to the Minimum Information for Publication of RT-qPCR Experiments (MIQE) guidelines (Bustin et al., 2009) using a LightCycler® 480 Real-Time PCR system (Roche Diagnostics, GmBh, Basa, Switzerland) following parameters described in Naidoo et al. (2013). The qBASEplus v1.0 (Biogazelle NV, Belgium) software package was used to perform normalization and relative quantification. Significance was determined using a two-tailed Student's *t*-test in Microsoft® Office Excel 2010.

#### Phylogenetic analysis

Protein sequences were retrieved from GenBank, Phytozome and JGI, and aligned using MUSCLE (Edgar, 2004). RAxML (Stamatakis, 2006) was used to search for the best scoring maximum likelihood tree with rapid bootstrapping, with GAMMA BLOSUM62 as an evolutionary model. A Bayesian inference analysis was conducted in MrBayes 3.2.2 (Ronquist and Huelsenbeck, 2003). Two chains were sampled once every thousand generations out of a total of one million generations. Trees were summarized with a 10% burn-in. All programs used were housed in Geneious software package version 7.1.5.

## Results

**Figure 1:**
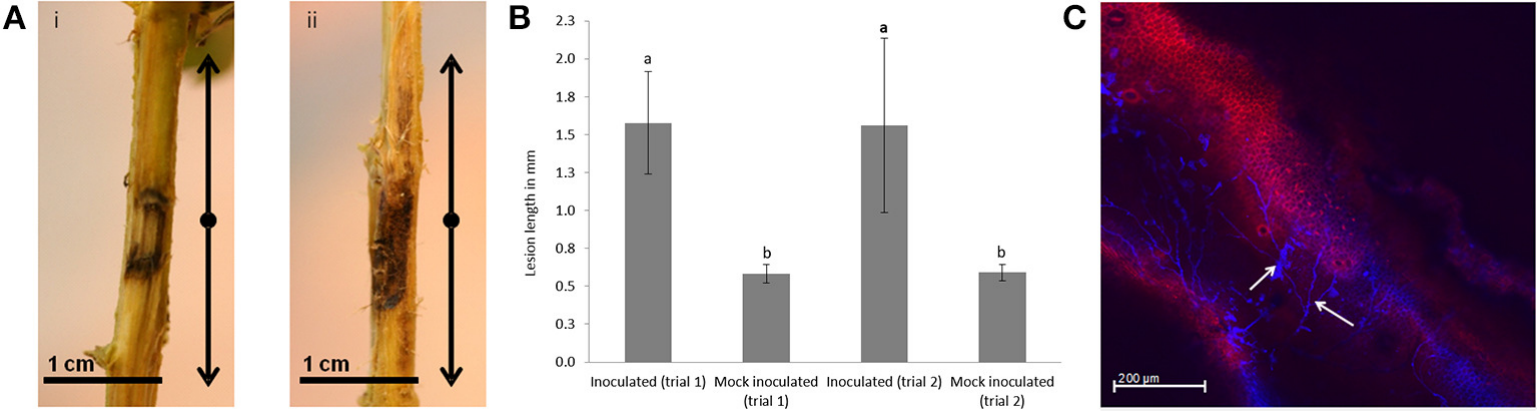
**Symptom development in *Eucalyptus nitens* following challenge with *Phytophthora cinnamomi*. (A)** A section of 1.5 cm stem tissue was harvested below and above the site of inoculation at 5 dpi. **(i)** Mock-inoculated and **(ii)** inoculated. **(B)** Lesions on *E. nitens* seedlings 6 wpi with *P. cinnamomi*. The small letters indicate that lesions on inoculated seedlings were significantly larger than the mock-inoculated negative control at *p* < 0.05 using the Mann–Whitney test for non-parametric data. Error bars show standard error based on *n* = 12 replicates. **(C)** Confocal microscopy of a longitudinal stem section showing *P. cinnamomi* hyphae (white arrows) at 4 dpi.

*P. cinnamomi* infected tissue for RNA-seq profiling was obtained by inoculating the stems of *E. nitens* seedlings and harvesting at 5 dpi. At this time-point, slight browning was visible around inoculation sites of control plants, whereas lesions of inoculated plants extended to the boundaries of the 3 cm sampled area (Figure 1A). At 6 wpi lesion length was pronounced compared to the mock-inoculated controls (Figure 1B). At 4 wpi, ~50% of the inoculated plants showed mortality. Lesion length was measured for live plants only. The efficacy of inoculation was verified by the presence of hyphae in stem tissue and lesion development over a 4 day time-course (Figure 1C).

### RNA-sequencing and mapping to *Phytophthora cinnamomi* and *Eucalyptus grandis* genomes

Approximately 36 million reads were obtained per sample, and reads were mapped to the *E. grandis* genome version 1.1 (Table 2). In addition to host transcripts, we mapped *P. cinnamomi* transcripts expressed *in planta*, based on the draft *P. cinnamomi* assembly. A low percentage of mapping (0.08%) was observed in the control (mock inoculated) samples, which were considered conserved eukaryotic gene sequences. On average, 1% of the reads mapped to the *P. cinnamomi* genome in the inoculated samples. Approximately 78% of the transcripts derived from both inoculated and mock-inoculated *E. nitens* samples mapped to the *E. grandis* genome (Table 2). The number of expressed genes and the average FPKM values were similar across the three biological replicates of each treatment which provided confidence that the samples were treated in a consistent manner and the results were comparable across data sets (Table 2).

**Table 2:** **Flagstat and FastQC RNA-seq mapping statistics of *Eucalyptus nitens* reads to the v1.1 *Eucalyptus grandis* and *Phytophthora cinnamomi* var *cinnamomi* genomes**.

**Sample name**	**Total reads mapped to *E. grandis***	**Properly paired to *E. grandis* (%)^a^**	**Singletons mapped to *E. grandis* (%)^b^**	**% GC content**	**Expressed genes in host**	**Average FPKM in host**	**Total reads mapped to pathogen**	**% reads mapped to pathogen**
Control 1	37444809	66.44	10.24	50	29024	493598	30756	0.08
Control 2	36111678	68.02	10.16	50	29250	492972	29274	0.08
Control 3	37060251	68.76	8.90	49	29135	467006	29116	0.08
Inoculated 1	37234371	66.13	11.65	49	29429	471923	444935	1.19
Inoculated 2	36622434	67.30	12.48	49	29407	497171	552202	1.51
Inoculated 3	36022978	68.19	10.18	49	29576	473466	196534	0.55

a *Number of proper pairs in proportion to the total reads mapped*.

b *Number reads where one from a pair in proportion to the total mapped*.

### *Phytophthora cinnamomi* genes expressed in planta

**Figure 2:**
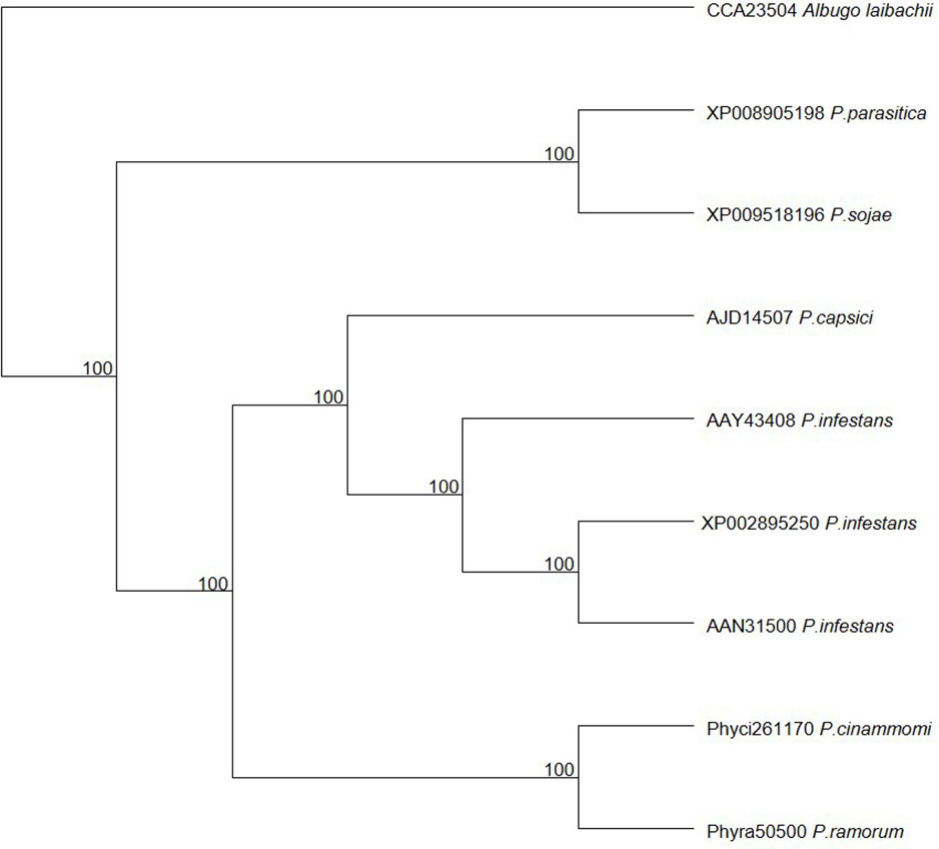
**Maximum likelihood phylogenetic tree of the putative *Phytophthora cinnamomi* crinkler (CRN) protein in relation to CRN proteins in other *Phytophthora* species**.

The list of transcripts expressed in the three inoculated *E. nitens* samples with CV < 0.2 are provided as a Supplementary File (Table S1). Of these 283 genes, the genes with hits (*E*-value ~0) to the plant host interactions database described as loss in pathogenicity, avirulence determinants, or reduced virulence are indicated in Table 3. Several of these have homology to transcripts with known roles in pathogenicity and virulence based on functional genetics experiments in other pathogen species. The highest expressed gene was a member of the CRN family protein. The *P. cinnamomi* putative CRN (JGI: Phyci261170) was aligned with other putative and described CRN family members and a maximum likelihood phylogenetic tree describing the relationship was produced. A congruent topology was recovered from Bayesian inference. The closest relationship was to a putative CRN protein from *P. ramorum*. Both the *P. ramorum* and *P. cinammomi* putative CRN proteins are closely related to the better characterized *P. infestans* CRN proteins (Figure 2).

**Table 3:** ***Phytophthora cinnamomi* genes expressed *in planta* implicated in pathogenicity or virulence (*E*-value < 0.01) based on comparison to the plant-host interactions database**.

**Gene identity**	**Average FPKM**	**Gene ontology**	***E*-value**	**Gene name**	**Knock-out phenotype**	**PHI base accession**
e_gw1.822.3.1	1443.83	gi|301096130|ref|XP_002897163.1|Crinkler (CRN) family protein	3.85E-91	crn1	Effector plant avirulence determinant	656
estExt_fgenesh1_pg.C_680034	1323.48	hydrogen-transporting ATPase activity, rotational mechanism	9.04E-26	invC	Reduced virulence	645
estExt_Genewise1.C_2370044	1096.91	DNA binding	5.16E-11	GzLam002	Reduced virulence	1533
e_gw1.40.157.1	950	zinc ion binding, glutathione peroxidase activity, response to oxidative stress	8.14E-35	MoHYR1	Reduced virulence	2356
fgenesh1_kg.2_#_22_#_Locus405v1rpkm794.38	915.58	protein binding, transcription factor binding, GTP binding, ATP binding	1.40E-67	CLPT1	Reduced virulence	339
e_gw1.76.61.1	853.95	catalytic activity, hydrolase activity, ATP binding, ATPase activity, ATPase activity	7.85E-55	PMR1	Reduced virulence	440
estExt_Genewise1.C_610064	741.59	protein kinase activity, GTP binding, protein-tyrosine kinase activity	1.27E-22	MoSNF1	Reduced virulence	1058
fgenesh1_kg.16_#_102_#_Locus840v1rpkm311.24	731.62	catalytic activity, FAD binding, oxidoreductase activity	3.59E-04	ALO1	Reduced virulence	197
fgenesh1_kg.9_#_117_#_Locus4954v1rpkm35.55	647.81	catalytic activity, cofactor binding, oxidoreductase activity	4.47E-32	MGG	Reduced virulence	881
fgenesh1_pg.124_#_4	620.76	FAD binding, oxidoreductase activity, cell redox homeostasis, electron transport	7.82E-06	SID1	Reduced virulence	1010
gm1.2704_g	421.24	catalytic activity, ATP binding, metabolism	0.01	ACL2	Loss of pathogenicity	2387
gm1.12073_g	324.79	nucleoside triphosphatase activity, nucleotide binding, hydrolase activity	2.38E-66	PEX6	Loss of pathogenicity	226
gm1.7056_g	315.86	catalytic activity, metabolism	6.30E-17	SidI	Reduced virulence	2321
gm1.272_g	294.34	catalytic activity, acetate-CoA ligase activity, AMP binding, etabolism	3.33E-22	AKT1	Loss of pathogenicity	133
e_gw1.31.72.1	268.44	microtubule motor activity, ATP binding, microtubule-based movement	2.50E-32	KIN2	Reduced virulence	465
e_gw1.1.234.1	254.89	catalytic activity, metabolism	9.49E-12	SidI	Reduced virulence	2321
e_gw1.93.22.1	254.29	protein binding, protein kinase activity, protein-tyrosine kinase activity	5.50E-22	Ste11	Loss of pathogenicity	2484
e_gw1.1.500.1	204.35	antioxidant activity, oxidoreductase activity	5.61E-04	TSA1	Reduced virulence	386
estExt_Genemark1.C_2810025	203.63	electron-transferring-flavoprotein dehydrogenase activity, electron transport	0	SIDA	Loss of pathogenicity	486
e_gw1.67.108.1	199.31	ATP binding	1.08E-57	LHS1	Reduced virulence	2058
e_gw1.82.257.1	198.02	ATP binding	3.65E-13	LHS1	Reduced virulence	2058
e_gw1.108.166.1	195.13	protein kinase activity, protein-tyrosine kinase activity	2.83E-66	MoCMK1	Reduced virulence	2158
e_gw1.2.24.1	187.23	helicase activity, nucleic acid binding, ATP dependent helicase activity, ATP binding	9.73E-70	VAD1	Reduced virulence	423
e_gw1.184.44.1	185.29	protein kinase activity, protein-tyrosine kinase activity, protein serine/threonine kinase activity	1.67E-51	SNF1	Reduced virulence	188
fgenesh1_pg.86_#_13	184.79	catalytic activity, aspartic-type endopeptidase activity, metabolism	1.15E-31	SidI	Reduced virulence	2321
MIX7251_264_83	180.35	polygalacturonase activity, carbohydrate metabolism	0	Pcipg2	Reduced virulence	2343
gm1.8946_g	164.67	ATP binding, nucleotide binding, nucleoside triphosphatase activity, tRNA ligase activity	2.70E-47	ABC4	Loss of pathogenicity	2067
e_gw1.2.738.1	153.76	hydrolase activity, cellulose binding, serine-type endopeptidase activity, blood coagulation	1.21E-46	CBEL	Effector plant avirulence determinant	660
fgenesh1_pg.112_#_14	142.08	gi|325187184|emb|CCA21725.1|bromodomain containing 2 putative [Albugo laibachii Nc14]	1.63E-09	GzBrom002	Reduced virulence	1317
e_gw1.11.45.1	137.55	phosphotransferase activity, alcohol group as acceptor	1.02E-31	VPS34	Loss of pathogenicity	195
e_gw1.74.48.1	116.37	protein binding, transcription factor binding, GTP binding, ATP binding, GTPase activity	5.62E-40	CLPT1	Reduced virulence	339
estExt_fgenesh1_pg.C_1470005	110.97	transporter activity, binding, ATPase activity, ATP binding, transport	1.15E-26	MgAtr4	Reduced virulence	310
gm1.14921_g	103.14	motor activity, ATP binding, myosin	3.16E-114	GzWing020	Reduced virulence	1648

### Differentially expressed genes in *Eucalyptus nitens*

**Figure 3:**
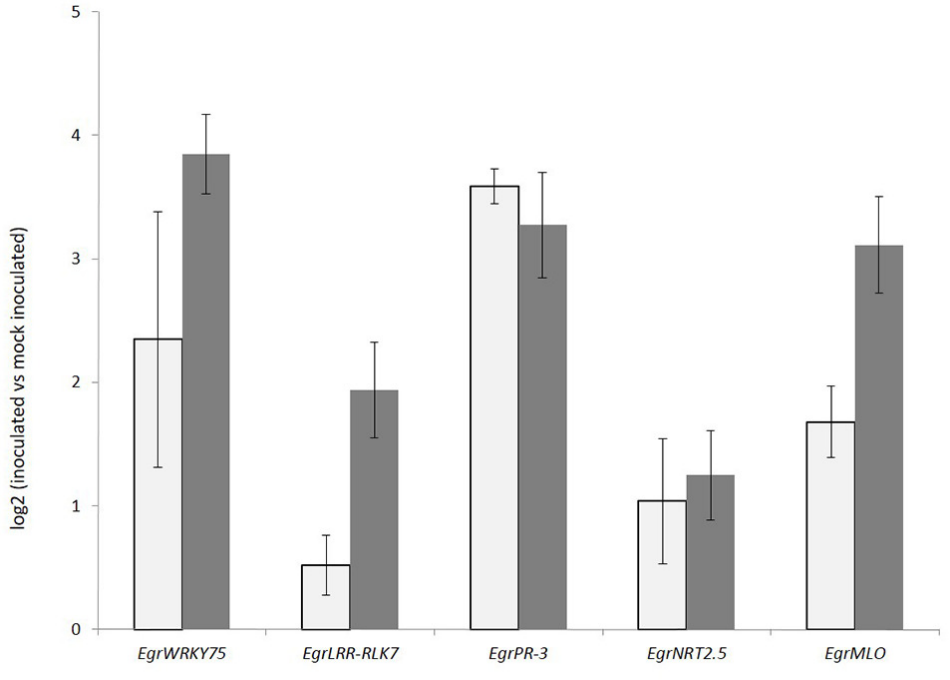
**Expression validation of selected genes in *Eucalyptus nitens* under *Phytophthora cinnamomi* inoculated compared to mock inoculated conditions 5 days post inoculation**. Gray bars represent RT-qPCR expression patterns and dark gray bars, RNA-seq expression patterns.

After mapping to the genome, Cuffdiff was used to compare pathogen-inoculated *E. nitens* samples with the mock-inoculated controls. A total of 890 up-regulated and 585 down-regulated gene models were observed at a false discovery rate (FDR) of 0.05. The expression of a sub-set of differentially expressed genes *EgrWRKY75, EgrPR-3, EgrNRT2.5, EgrMLO*, and *EgrLRR-RLK7* were validated using RT-qPCR (Figure 3). The tissue for the RT-qPCR validation was sourced from a separate trial that was set up identically to the first trial, with three biological repeats. The expression patterns were comparable to RNA-seq results, with the correlation coefficient between the RNA-seq and the RT-qPCR expression being 0.73.

### Over-represented gene ontologies

**Figure 4:**
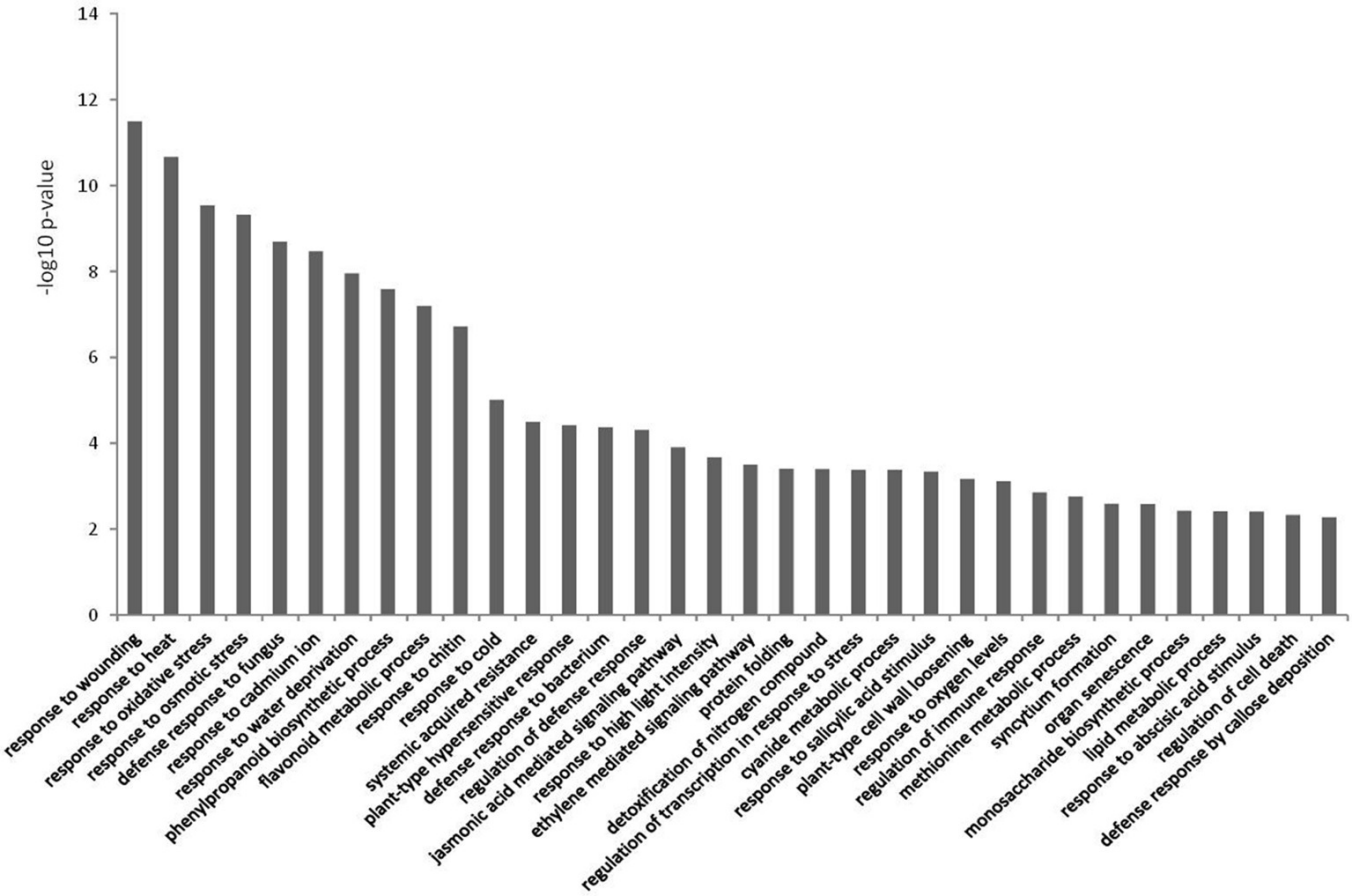
**Over-represented Gene Ontologies in the category biological process for the genes up-regulated in response to *Phytophthora cinnamomi* challenge in *Eucalyptus nitens* at 5 dpi**.

Differentially expressed genes with matching TAIR10 IDs were used in BiNGO to test for over-representation against the *A. thaliana* genome as background. In the up-regulated dataset for biological processes, the majority of over-represented GO terms were related to defense (Figure 4). Several of the categories involved JA and ET signaling, and there were a few terms related to SA (Figure 4 and Table S1). Phenylpropanoid pathway terms and aromatic compound synthesis related to flavonoid biosynthesis were also found in this dataset (Figure 4 and Table S2). Another prominent term in the up-regulated dataset was response to water deprivation/water stress terms.

**Figure 5:**
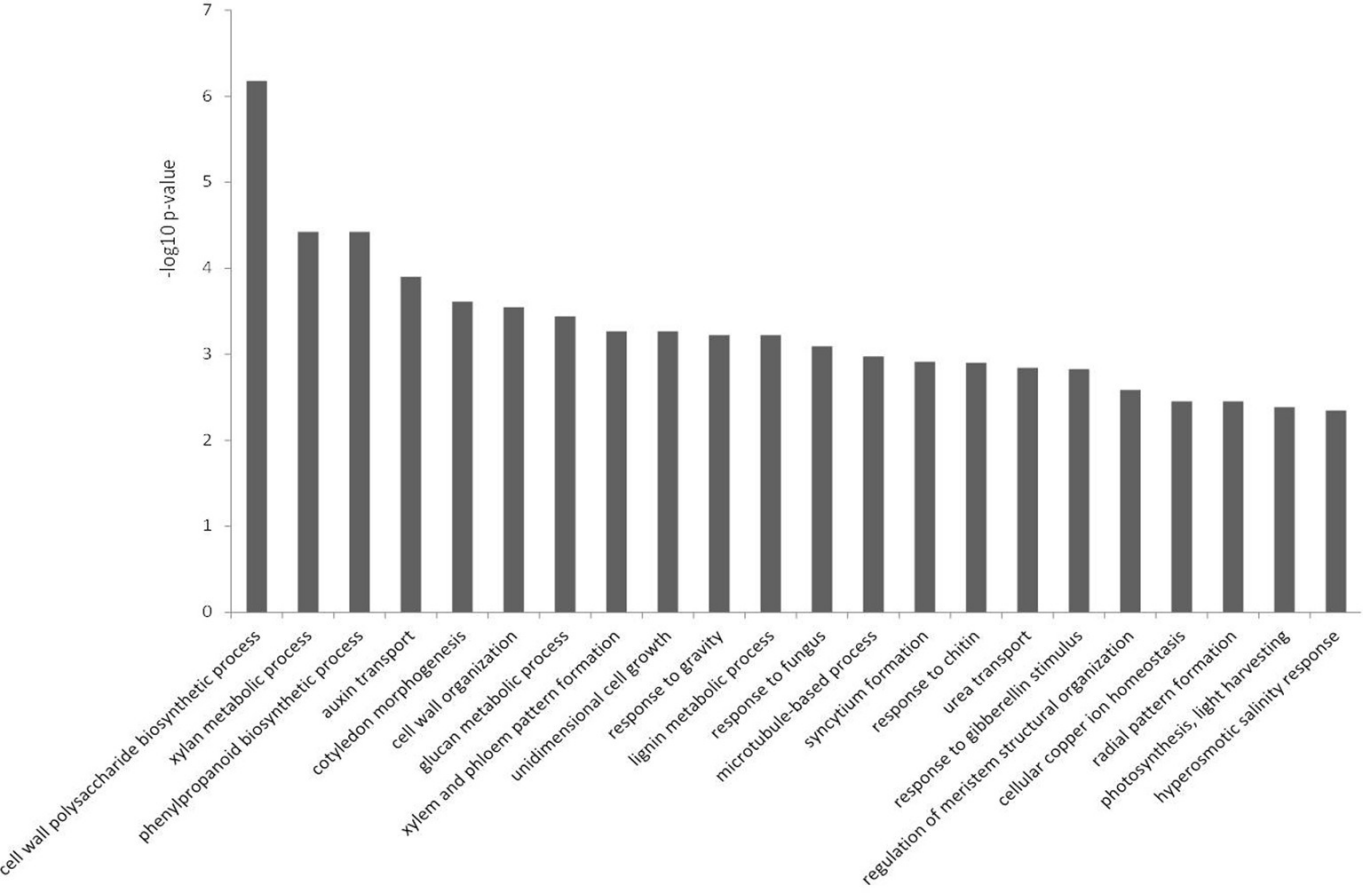
**Over-represented Gene Ontologies in the category biological process for the genes down-regulated in response to *Phytophthora cinnamomi* challenge in *Eucalyptus nitens* at 5 dpi**.

Over-represented GO terms in the down-regulated dataset for biological processes (Figure 5) were predominantly related to growth, cell wall modifications, and cell wall chemistry. The phenylpropanoid pathway terms were related to lignin biosynthesis, as opposed to flavonoid synthesis in the up-regulated dataset (Figure 5 and Table S2). There were minimal biotic stress-related terms, and hormone-related terms in this dataset such as auxin and gibberellin were apparent (Figure 5 and Table S1). Photosynthesis-related terms were also over-represented.

One of the noteworthy aspects of the RNA-seq data obtained was that several putative *PR* genes displayed particularly high fold-change values, mostly in the up-regulated dataset. This is summarized in Table 4. Within the *E. nitens*- *P. cinnamomi* interaction, the most prominent putative *PR* genes were *PR-1, PR-3* (chitinase), and *PR-5* (thaumatin-like and osmotin). These were not only consistently highly up-regulated in inoculated tissue, but there were multiple *E. nitens* putative orthologs per *A. thaliana* gene that are all regulated at similar fold-change levels. Putative *E. nitens* orthologs of *PR-4* (chitin-binding), *PR-8* (chitinase class III), and *PR-12* (defensins) were up-regulated. The *PR-9* (peroxidase) and *PR-10* (ribonuclease-like) classes contained a mix of up- and down-regulated putative orthologs. *PR-14* (lipid transfer proteins) and *PR-15* (oxalate oxidase/germin) putative orthologs were all down-regulated.

**Table 4:** **Expression of pathogenesis related genes in response to *Phytophthora cinnamomi* inoculation in *Eucalyptus nitens***.

**Gene family**	**TAIR ID**	***E. grandis* ID**	**Description**	**log^2^ expression**
*PR-1*	AT2G14580.1	Eucgr.D01552	Basic pathogenesis-related protein 1	4.56
	AT2G14580.1	Eucgr.G01140	Basic pathogenesis-related protein 1	4.40
	AT2G14580.1	Eucgr.G01148	Basic pathogenesis-related protein 1	4.37
	AT2G14580.1	Eucgr.D01560	Basic pathogenesis-related protein 1	4.36
	AT2G14580.1	Eucgr.G01171	Pathogenesis-related gene 1	4.31
	AT2G14610.1	Eucgr.G01134	Basic pathogenesis-related protein 1	3.34
	AT2G14610.1	Eucgr.G01137	Pathogenesis-related gene 1	3.24
	AT2G14610.1	Eucgr.L02505	Pathogenesis-related gene 1	3.14
	AT2G14610.1	Eucgr.L01707	Pathogenesis-related gene 1	3.13
*PR-3*—Chitinase class I, II, IV, VI, VII	AT3G12500.1	Eucgr.L00941	Basic chitinase	4.02
	AT3G12500.1	Eucgr.J02519	Basic chitinase	3.94
	AT3G12500.1	Eucgr.L00938	Basic chitinase	3.93
	AT3G54420.1	Eucgr.H00326	Homolog of carrot EP3-3 chitinase	3.75
	AT3G54420.1	Eucgr.H00321	Homolog of carrot EP3-3 chitinase	3.75
	AT3G12500.1	Eucgr.L00939	Basic chitinase	3.73
	AT3G54420.1	Eucgr.H00328	Homolog of carrot EP3-3 chitinase	3.57
	AT3G12500.1	Eucgr.L00937	Basic chitinase	3.47
	AT3G12500.1	Eucgr.I01495	Basic chitinase	3.11
	AT3G54420.1	Eucgr.K02166	Homolog of carrot EP3-3 chitinase	2.53
	AT3G54420.1	Eucgr.K02166	Homolog of carrot EP3-3 chitinase	2.21
	AT3G54420.1	Eucgr.A00020	Homolog of carrot EP3-3 chitinase	1.43
	AT1G05850.1	Eucgr.H00455	Chitinase family protein (TAIR 9)	−0.75
	AT3G16920.1	Eucgr.H04034	Chitinase-like protein 2	−1.33
*PR-4*—Chitin-binding	AT3G04720.1	Eucgr.B02124	Pathogenesis-related 4	3.42
	AT3G04720.1	Eucgr.L03258	Pathogenesis-related 4	3.32
	AT3G04720.1	Eucgr.B02122	Pathogenesis-related 4	3.06
*PR-5*—Thaumatin-like and osmotin	AT1G20030.2	Eucgr.E01382	Pathogenesis-related thaumatin superfam	5.42
	AT1G20030.2	Eucgr.E01384	Pathogenesis-related thaumatin superfam	5.31
	AT1G20030.2	Eucgr.E01389	Pathogenesis-related thaumatin superfam	5.23
	AT1G20030.2	Eucgr.E01385	Pathogenesis-related thaumatin superfam	5.13
	AT4G11650.1	Eucgr.H03863	Osmotin 34	5.09
	AT1G20030.2	Eucgr.E01381	Pathogenesis-related thaumatin superfam	4.96
	AT4G11650.1	Eucgr.H03865	Osmotin 34	4.93
	AT4G11650.1	Eucgr.H03864	Osmotin 34	4.77
	AT4G11650.1	Eucgr.L01962	Osmotin 34	4.69
	AT4G11650.1	Eucgr.E00557	Osmotin 34	4.43
	AT4G11650.1	Eucgr.D01888	Osmotin 34	3.57
	AT4G11650.1	Eucgr.D01892	Osmotin 34	3.34
	AT4G11650.8	Eucgr.D01887	Osmotin 34	3.31
	AT4G11650.9	Eucgr.E00560	Osmotin 34	2.97
	AT5G38280.1	Eucgr.A01474	PR5-like receptor kinase	1.03
	AT5G38280.1	Eucgr.A01470	PR5-like receptor kinase	0.96
	AT5G38280.1	Eucgr.A01478	PR5-like receptor kinase	0.78
	AT2G28790.1	Eucgr.J02061	Pathogenesis-related thaumatin superfam	−1.18
	AT4G38660.1	Eucgr.G01772	Pathogenesis-related thaumatin superfam	−1.33
	AT1G73620.1	Eucgr.B00944	Pathogenesis-related thaumatin superfam	−1.36
*PR-8*—Chitinase class III	AT5G24090.1	Eucgr.E00091	Chitinase A	2.40
	AT5G24090.1	Eucgr.L03478	Chitinase A	1.52
*PR-9*—Peroxidase	AT4G37530.1	Eucgr.J02352	Peroxidase superfamily protein	3.65
	AT4G11600.1	Eucgr.D01857	Glutathione peroxidase 6 (TAIR 9)	3.08
	AT1G71695.1	Eucgr.F04198	Peroxidase superfamily protein	2.34
	AT1G05260.1	Eucgr.A01385	Peroxidase superfamily protein	1.83
	AT1G71695.1	Eucgr.F04195	Peroxidase superfamily protein	1.46
	AT1G71695.1	Eucgr.L02740	Peroxidase superfamily protein	1.39
	AT5G40150.1	Eucgr.J02173	Peroxidase superfamily protein	−1.23
	AT5G42180.1	Eucgr.F03724	Peroxidase superfamily protein	−1.76
	**AT4G21960.1**	**Eucgr.E04056**	**Peroxidase superfamily protein**	**−1.85**
*PR-10*—Ribonuclease-like	AT1G80780.3	Eucgr.F03953	Polynucleotidyl transferase, ribonuclease	0.83
	AT5G22250.1	Eucgr.J00535	Polynucleotidyl transferase, ribonuclease	−1.46
*PR-12*—Defensins	AT4G11393.1	Eucgr.K03440	Defensin-like (DEFL) family protein	2.72
*PR-14*—Lipid transfer proteins	AT5G48485.1	Eucgr.H00727	Bifunctional inhibitor/lipid-transfer protein	−0.85
	AT5G64080.1	Eucgr.I02679	Bifunctional inhibitor/lipid-transfer protein	−0.95
	AT3G18280.1	Eucgr.B00824	Bifunctional inhibitor/lipid-transfer protein	−1.00
	AT5G59320.1	Eucgr.K01283	Lipid transfer protein 3	−1.20
	AT5G55460.1	Eucgr.F03514	Bifunctional inhibitor/lipid-transfer protein	−1.31
	AT5G59320.1	Eucgr.A00746	Lipid transfer protein 4	−1.60
	AT5G05960.1	Eucgr.K03041	Bifunctional inhibitor/lipid-transfer protein	−1.66
	AT5G59320.1	Eucgr.K01282	Lipid transfer protein 5	−1.76
*PR-15*—Oxalate oxidases (Germin)	AT3G62020.1	Eucgr.A00990	Germin-like protein 10	−1.18

*Up and down regulated genes are indicated as a gradient from bright red to bright green*.

**Figure 6:**
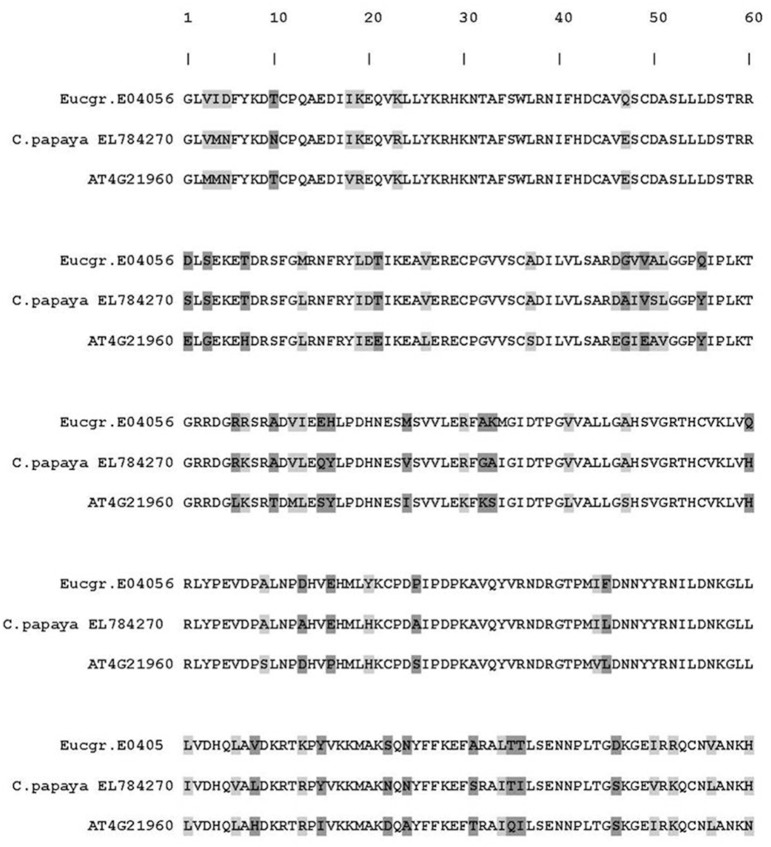
**Amino acid alignment of the putative PR-9 peroxidase ortholog of *Eucalyptus nitens* (Phytozome: Eucgr.E04056) in comparison to the *Arabidopsis thaliana* (TAIR number AT4G21960) and *Carica papaya* (Genbank: EL784270) orthologs**. Light gray highlights conservative amino acid substitutions and dark gray indicates non-conservative substitutions.

The *E. nitens* putative *PR-9* ortholog (Eucgr.E04056) was down regulated by a 1.85 fold-change on a log_2_ scale in the *P. cinnamomi*-inoculated tissue (indicated in bold in Table 4). The sequence of this ortholog was compared to peroxidase from *Carica papaya* (EL784270) and *A. thaliana*. The amino acid alignment is indicated in Figure 6. The *E. nitens PR-9* sequence shared 80% amino acid identity with the *C. papaya PR-9* sequence.

## Discussion

The compatible interaction between *E. nitens* and *P. cinnamomi* provided a model system to study the compatible interaction between *Eucalyptus* spp. and *Phytophthora* spp. One year old *E. nitens* plants were stem inoculated using *P. cinnamomi* and pronounced lesions were obtained (Figure 1) suggesting that successful infection had occurred. This was corroborated with the presence of hyphae in the stems as early as 48 hpi in some instances and subsequently consistently observed at 5 dpi. In order to gain a better understanding of this compatible pathosystem, a dual RNA sequencing approach, as described previously for other plant-pathogen interactions (Hayden et al., 2014), was undertaken to concurrently detect pathogenicity factors and host responses.

Analysis of transcripts mapping to the *P. cinnamomi* genome confirmed the presence of ~1% pathogen in the tissue profiled. We obtained 78% mapping of the *E. nitens* transcripts to the *E. grandis* genome which was similar to that obtained in a study by Ward and Weber (2012) where raspberry transcripts were mapped to the strawberry genome. This substantiates the use of cross-species resources in the event that no such genomic resources are available for the species of interest.

Table 3 indicates possible determinants expressed *in planta* which may contribute to the susceptible outcome of the interaction. A pathogen transcript encoding a CRN family protein was highly expressed *in planta*. In *Phytophthora* species, the CRNs are a complex family of large proteins and various experiments suggest that some CRNs are able to target host factors to suppress plant defenses (Adhikari et al., 2013). The *P. cinnamomi* CRN is closely related to the CRN1 protein from *P. infestans*, suggesting that it may have the same role as described in *P. infestans*. Torto et al. (2003) showed that the *P. infestans* CRN1 and CRN2 effectors were expressed during infection of tomato and that CRN1 and CRN2 were able to cause necrosis in tobacco and CRN2 induced PR1a expression in tomato. While some CRN effectors are known to target host nuclei (Stam et al., 2013a) the role in virulence may be diverse in various life stages of *P. infestans* (Resjö et al., 2014). Liu et al. (2011) described two CRN effectors from *P. sojae* where one induced cell death and the other suppressed cell death in soybean. More recently, it was shown that the two effectors together, or one alone, can suppress host defenses by interacting with catalases and in so doing, modulate the HR and H_2_O_2_ levels *in planta* (Zhang et al., 2015). Another well characterized example of an apoplastic effector is that of the *Cellulose Binding, Elicitor, and Lectin-like* (*CBEL*) transcript, which elicits necrosis and defense gene expression in hosts and is necessary for attachment onto plant surfaces (Mateos et al., 1997; Séjalon-Delmas et al., 1997). Further examination of the virulence factors and the host targets they affect, will provide insight into the cause of the host responses observed in *E. nitens*.

The over-represented GO terms in *E. nitens* challenged with *P. cinnamomi* (Figures 4, 5) were indicative of a host actively attempting to combat infection, albeit at a late stage of a compatible interaction. We observed differential expression of various defense responses, but highlight two possible factors contributing to susceptibility.

Some physiological responses to *P. cinnamomi* include the down-regulation of photosynthesis-related terms and up-regulation of water stress terms. Various tree species inoculated with *P. cinnamomi* show declines in stomatal conductance and photosynthesis. In *Eucalyptus sieberi*, this decline is associated with susceptibility, since these factors decreased less severely in resistant *Eucalyptus sideroxylon* (Dempsey et al., 2012). In *Quercus suber*, photosynthesis and stomatal conductance also decreased after inoculation (Medeira et al., 2012). Manter et al. (2007) noted the prevalence of photosynthetic and stomatal conductance decreases in *Phytophthora*-host interactions. They showed that photosynthetic decline could be caused by elicitins in the absence of water stress. Maintaining adequate photosynthetic levels may assist tolerance or resistance, which is a possible explanation of why lower photosynthetic rates and stomatal conductance is associated with an increase in pathogen (Portz et al., 2007) and lower tolerance to *Phytophthora* spp. (Reeksting et al., 2014).

Distinct components of the phenylpropanoid pathway are present in both the up- and down-regulated datasets. There are several up-regulated genes with GO annotations associated with flavonoid biosynthesis. Susceptible *Lupinus angustifolius* up-regulated the flavonoid genistein in response to *P. cinnamomi* (Gunning et al., 2013) however, certain *Citrus* flavonoids have an antimicrobial action against *Phytophthora citrophthora* (del Río et al., 2004). In the down-regulated dataset, GO terms associated with lignin synthesis via the phenylpropanoid pathway are over-represented. For lignin synthesis, most genes encoding enzymes involved in biosynthesis of the coniferyl alcohol (G subunit) and sinapyl alcohol (S subunit) are down-regulated, although genes encoding enzymes catalyzing the synthesis of coumaryl alcohol (H subunit) are up-regulated (Table S2). Since synthesis of the S and G subunits is possibly suppressed, lignin biosynthesis could be down-regulated in *E. nitens*. Lignin is associated with strengthening of cell-walls and helps prevent penetration by a pathogen (Bechinger et al., 1999), and down-regulation of monolignols can compromise host resistance (Naoumkina et al., 2010). Lignin synthesis plays a role in raspberry responses to challenge with *P. rubi* (Ward and Weber, 2012) and transgenic potato plants with limited phenylpropanoid substrates had increased susceptibility to *P. infestans* (Yao et al., 1995).

Gene ontology terms pertaining to JA, SA, and ET pathways were over-represented in the up-regulated dataset in *E. nitens*, suggesting that they could play a role in defense signaling for this interaction. Other studies involving hosts inoculated with *Phytophthora* spp. have also shown mixed hormone responses (Attard et al., 2010; Shibata et al., 2010). JA may be needed for successful defense against *P. cinnamomi* in maize, a resistant monocot (Allardyce et al., 2013). Terms related to GA and auxin were over-represented in the down-regulated dataset. Treatment of soybean with GA increased susceptibility to *P. sojae* (Sugano et al., 2013) and it has been proposed that GA influences defense against necrotrophic fungi by repressing resistance (Mengiste, 2012). The role of auxin in defense against *P. cinnamomi* has recently been described (Eshraghi et al., 2014b), where *Arabidopsis* auxin sensitivity and transport mutants were shown to be highly susceptible to the pathogen. Treatment of *L. angustifolius* with an inhibitor of auxin transport increased susceptibility to *P. cinnamomi*.

Since *PR* genes are markers of defense hormone signaling, the different putative *PR* genes expressed reflect the mix of JA and SA signaling noted in the over-representation analysis.

Gene models identified as *PR* genes in this dataset are putative orthologs of *A. thaliana PR* genes. For many of these genes, there are multiple differentially expressed *E. grandis* gene models matching to one *A. thaliana* putative ortholog and an expansion of several *PR* genes in *E. grandis* has been noted (Naidoo et al., 2014). Expression of these multiple *PR* gene transcripts in *E. nitens* could indicate that some of the orthologs have slightly different functions and are all used during a defense response.

Transcription of *PR-1*, chitinase (*PR-3*), chitin-binding protein (*PR-4*), and thaumatin-like protein/osmotin (*PR-5*) putative orthologs appears to be highly up-regulated in *E. nitens* (Table 4). A *Phytophthora*-resistant potato expresses *PR-1* constitutively (Ali et al., 2012) and constitutive expression of *PR-1* in transgenic tobacco confers resistance to *P. parasitica* (Alexander et al., 1993). Transgenic plants over-expressing *PR-5* genes have been shown to increase resistance to *P. citrophthora* and *P. infestans* (Fagoaga et al., 2001; Pushin et al., 2010; Acharya et al., 2013).

Peroxidases (PR-9) are potential cross-species *Phytophthora* effector targets, since a certain *C. papaya* peroxidase (EL784270) and its putative orthologs have been suppressed in different hosts upon inoculation with *P. sojae, P. palmivora*, and *P. infestans* (Moy et al., 2004; Restrepo et al., 2005; Porter et al., 2009). An *E. grandis* gene, Eucgr.E04056, is highly similar to the *C. papaya* ortholog, and is also strongly suppressed in the current interaction. Putative *PR-14* and *PR-15* orthologs were down-regulated in *E. nitens* and while these orthologs would have to be characterized further, it is tempting to speculate that their suppression may be driving susceptibility. For example, enhanced resistance to *P. nicotianae* was conferred by a pepper lipid transfer protein over-expressed in tobacco (Sarowar et al., 2009) and *PR-15* may encode for a germin-like oxalate oxidase known to produce hydrogen peroxide that is toxic to pathogens (van Loon et al., 2006; Ferreira et al., 2007).

## Conclusion

The outcomes of this dual RNA-seq study provided valuable insights into *P. cinnamomi* pathogenicity and virulence factors and *E. nitens* defense mechanisms utilized against *P. cinnamomi*. Several factors may contribute to the compatibility however, we have used further sequence and functional genetics support to motivate that the *P. cinnamomi CRN* and the *E. nitens PR-9* genes are important contributors to the susceptible outcome. Future work involving comparison with a resistant interaction over a time-course is required to provide an indication of host targets manipulated by *P. cinnamomi* and to enhance understanding of the defense pathways required for resistance.

## Author contributions

FM, LS, SiN, and TM performed the experimental work, conducted and interpreted data analyses. SaN conceived the study, obtained funding to support the research. AM provided input into the experimental design and technical aspects of RNA-sequencing and assisted with critical evaluation of the manuscript. FM, LS, and SaN wrote the manuscript with input from DB and NV, who supervised aspects of this research.

### Conflict of interest statement

The authors declare that the research was conducted in the absence of any commercial or financial relationships that could be construed as a potential conflict of interest.
